# Malate enhances survival of zebrafish against *Vibrio alginolyticus* infection in the same manner as taurine

**DOI:** 10.1080/21505594.2020.1750123

**Published:** 2020-04-21

**Authors:** Man-Jun Yang, Di Xu, Dai-Xiao Yang, Lu Li, Xuan-Xian Peng, Zhuang-gui Chen, Hui Li

**Affiliations:** a Center for Proteomics and Metabolomics, State Key Laboratory of Biocontrol, Guangdong Province Key Laboratory for Pharmaceutical Functional Genes, School of Life Sciences, Sun Yat-sen University, University City, Guangzhou, People’s Republic of China; b Laboratory for Marine Fisheries Science and Food Production Processes, National Laboratory for Marine Science and Technology, Qingdao, China; c Tibet Vocational Technical College, Lhasha, People’s Republic of China; d Third Affiliated Hospital of Sun Yat-sen University, Guangzhou 510630, People’s Republic of China

**Keywords:** Taurine, malate, reprogramming metabolomics, *Vibrio alginolyticus*, aquaculture, zebrafish

## Abstract

Development of low-cost and eco-friendly approaches to fight bacterial pathogens is especially needed in aquaculture. We previously showed that exogenous malate reprograms zebrafish’s metabolome to potentiate zebrafish survival against *Vibrio alginolyticus* infection. However, the underlying mechanism is unknown. Here, we use GC-MS based metabolomics to identify the malate-triggered metabolic shift. An activated TCA cycle and elevated taurine are identified as the key metabolic pathways and the most crucial biomarker of the reprogrammed metabolome, respectively. Taurine elevation is attributed to the activated TCA cycle, which is further supported by the increased expression of genes in the metabolic pathway of taurine biosynthesis from the isocitrate of the TCA cycle to taurine. Exogenous taurine increases the survival of zebrafish against *V. alginolyticus* infection as malate did. Moreover, exogenous taurine and malate regulate the expression of innate immunity genes and promote the generation of reactive oxygen species and nitrogen oxide in a similar way. The two metabolites can alleviate the excessive immune response to bacterial challenge, which protects fish from bacterial infection. These results indicate that malate enhances the survival of zebrafish to *V. alginolyticus* infection via taurine. Thus, our study highlights a metabolic approach to enhance a host’s ability to fight bacterial infection.

## Introduction

*Vibrio alginolyticus* is responsible for fatal illnesses such as gastroenteritis, septicemia, and necrotizing fasciitis in humans [1,2], acute hepatopancreatic necrosis in shrimp [3], and lethargy, erratic swimming, excessive mucus production, rotten fins, congestion of livers and kidneys, and enlargement of spleens in fish [4,5]. These shrimp and fish diseases cause severe economic losses and consequently preclude the sustainability of this industry [3,5]. Vaccination is an effective measure to deal with *V. alginolyticus* infection [6,7], which belongs to the low-cost and “green” approaches against bacterial pathogens [8]. Despite the advantages of vaccination, the approach is not applicable to small fish that are frequently raised in developing countries like China. Besides vaccine, antibiotics are the major approach to treat bacterial infection in aquaculture [9]; however, the overuse and misuse of antibiotics have resulted in the emergence of multidrug-resistant bacteria, microorganism substitution, ecological, and public health impacts [10,11].

The recently developed metabolome-reprogramming is an effective low-cost and “green” approach. It is performed by comparison between a “normal metabolome” and a “changed metabolome” to identify crucial metabolites as biomarkers. Then, the key biomarkers are used to reprogram the changed metabolome to a normal-like metabolome, which leads to changes in biological phenotypes [12,13]. This is because organisms can regulate their metabolomes to cope with internal and external stresses [14–18]. Thus, susceptible and less susceptible hosts have infective and anti-infective metabolomes, respectively [19,20]. Comparisons between the two differential metabolomes identify myo-inositol, L-leucine, N-acetylglucosamine, phenylalanine, glucose, L-proline, and palmitic acid as reprogramming metabolites of the infective metabolomes [20–22]. These reprogramming molecules reprogram infective metabolomes to anti-infective metabolomes. Therefore, susceptible hosts acquire resistance to bacterial infection [23–27]. Using this approach, we showed that elevated and decreased levels of malate are detected, respectively, in the survival and death of zebrafish infected with *V. alginolyticus*. The metabolite was identified as the most crucial biomarker that differentiates the infective metabolome from the anti-infective metabolome. Exogenous malate potentiated zebrafish to combat the bacterium and thereby elevated zebrafish survival [28]. However, how malate reprograms the infective metabolome and the relationship between the reprogramming and innate immune response are unknown.

Here, comparative metabolomics was used to explore the mechanisms by which the malate reprogramed zebrafish (*Danio rerio*) metabolome fights infection caused by *V. alginolyticus*. Elevated taurine was identified as the crucial biomarker of the reprogrammed metabolome. Exogenous taurine increased the survival of zebrafish infected with *V. alginolyticus* via regulating innate immunity, NO, ROS, glutathione peroxidase, and phagocytosis in a manner similar to malate. These results are described below.

## Results

### Malate reprograms the metabolome of zebrafish

To investigate how malate reprograms the zebrafish metabolome to contribute to their survival against *V. alginolyticus* infection, humoral fluid was used for GC-MS-based metabolomics in the reprogramming group and control group. Representative total ion current chromatograms were shown in Figure S1(a). After the removal of the internal standard ribitol, any known artificial peaks, and merge of the same compounds, 108 metabolites with reliable signals were identified in each sample. Metabolic profiles of the control and experimental groups were displayed as a heatmap in Figure S1(b). Five samples with two technical repeats for each sample were carried out in each group, yielding 20 data sets. The correlation coefficient between technical replicates varied between 0.9979 and 0.9999, demonstrating the reproducibility of the data (Figure S1(c)). According to the KEGG (http://www.kegg.jp/) annotation and NCBI PubChem (https://pubchem.ncbi.nlm.nih.gov/), the metabolites were classified into five categories, carbohydrates (27.78%), amino acids (22.22%), nucleotides (12.04%), fatty acids (20.37%), and others (17.59%) (Figure S1(d)).

**Figure 1.**
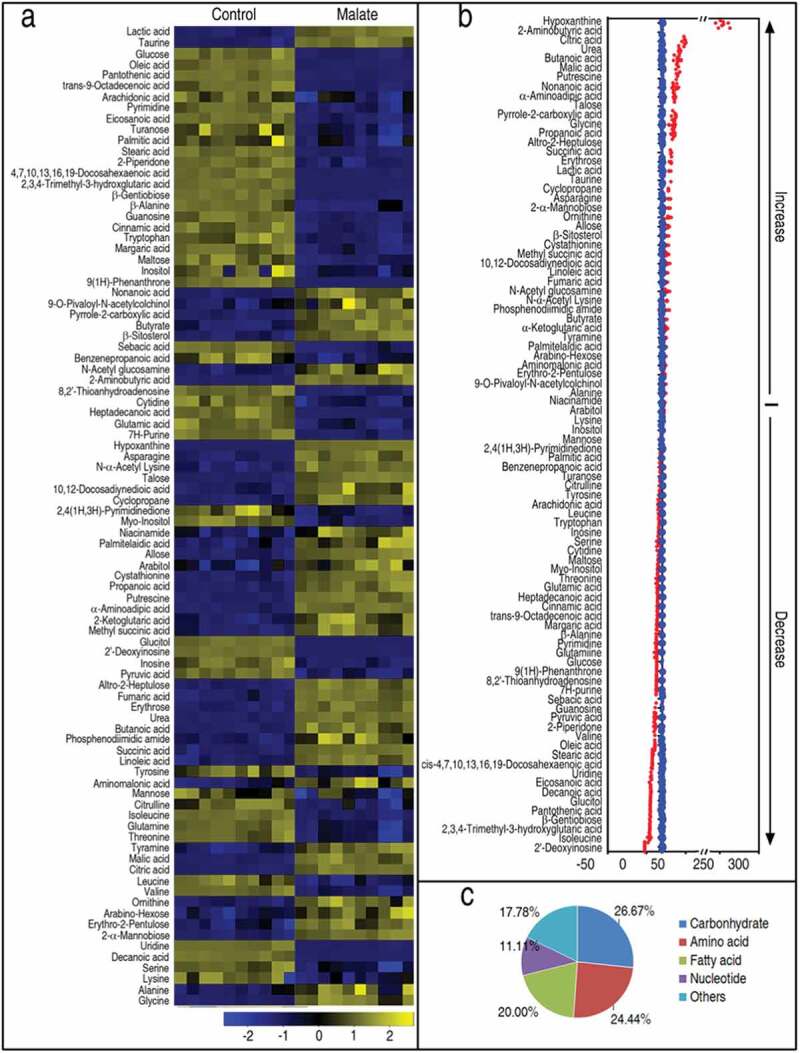
Differential metabolomic profiling in malate group in response to *V. alginolyticus* infection. Zebrafish were injected with and without 70 μg malate as malate group and control group, respectively, five for each group. Humoral fluid was collected for GC-MS analysis. (a) Heat map showing differential 90 metabolites. Yellow and blue indicate increase and decrease of metabolites relative to the median metabolite level of the control, respectively (see color scale). (b) Z-score plot of differential metabolites based on control. Each point represents one metabolite in one technical repeat and colored by sample types. (c) Category of 90 differential abundance of metabolites.

To identify malate-triggered metabolic features that distinguish the reprogramming group from the control group, a two-sided Mann–Whitney U test coupled with a permutation test was used to identify the metabolites of differential abundance between the two groups. Using the Mann–Whitney U test, 90 metabolites of differential abundance were identified (*p *< 0.01), which corresponded to a false discovery rate (FDR) of 0.0445%. These differential metabolites are shown in Figure 1(a) as a heatmap. The Z-score plot ranged from −31.89 to 334.21 in the reprogramming group. In the reprogramming group, the abundance of 43 metabolites was increased, and that of 47 metabolites was decreased as compared with the control group (Figure 1(b)). The 90 metabolites were categorized to 26.67% carbohydrates (24/90), 24.44% amino acids (22/90), 20.0% fatty acids (18/90), 11.11% nucleotides (10/90), and 17.78% others (16/90) (Figure 1(c)). These results suggest exogenous malate causes a metabolic shift in zebrafish that may promote their survival against *V. alginolyticus* infection.

### Enrichment of pathways in the malate-reprogramming metabolome

**Figure 2.**
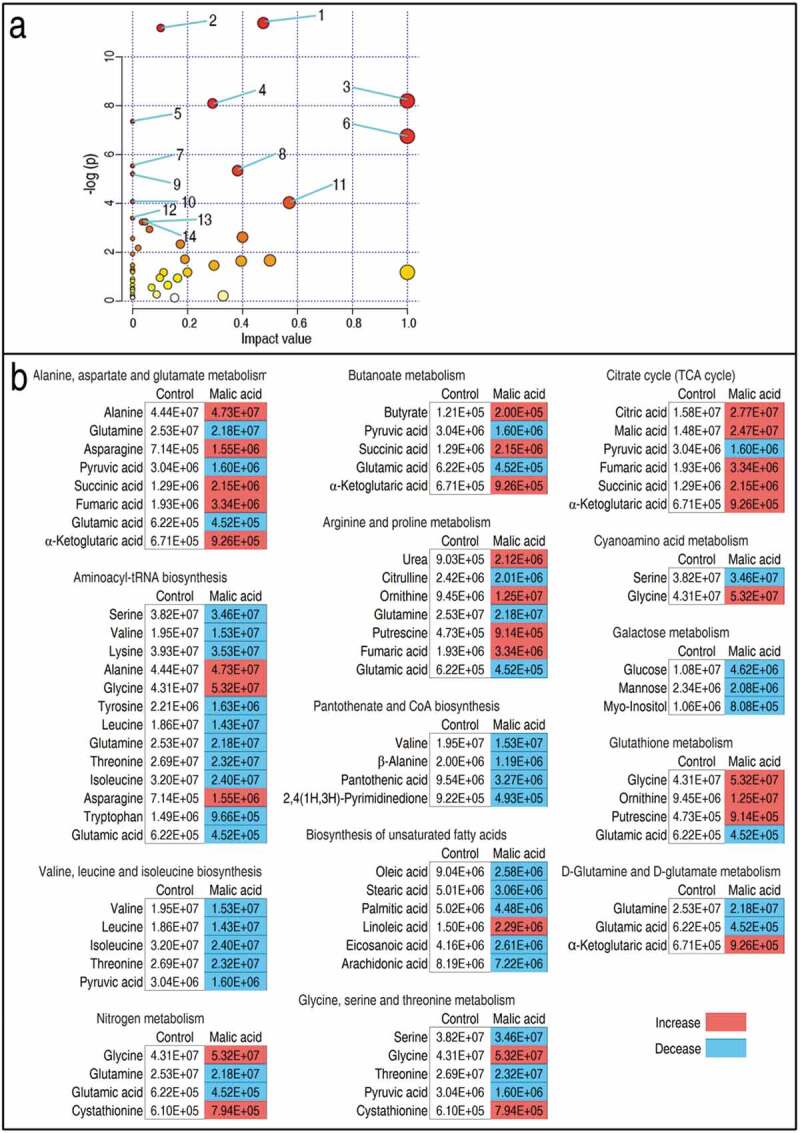
Pathway enrichment and analysis. (a) Pathway enrichment of varied metabolites in the malic acid group. Significantly enriched pathways are selected to be plotted based on raw p. 1, Alanine, aspartate, and glutamate metabolism; 2, Aminoacyl-tRNA biosynthesis; 3, Valine, leucine, and isoleucine biosynthesis; 4, Citrate cycle (TCA cycle); 5, Nitrogen metabolism; 6, D-Glutamine and D-glutamate metabolism; 7, Butanoate metabolism; 8, Arginine and proline metabolism; 9, Pantothenate and CoA biosynthesis; 10, Biosynthesis of unsaturated fatty acids; 11, Glycine, serine, and threonine metabolism; 12, Cyanoamino acid metabolism; 13, Glutathione metabolism; 14, Galactose metabolism. (b) integrative analysis of metabolites in significantly enriched pathways. Red and blue indicate increased and decreased metabolites, respectively.

We further investigated the pathways that were affected by exogenous malate, and 14 metabolic pathways were enriched. According to the impact (weight), they were ranked from high to low as follows: alanine, aspartate and glutamate metabolism > aminoacyl-tRNA biosynthesis > valine, leucine and isoleucine biosynthesis > citrate cycle (TCA cycle) > nitrogen metabolism > D-glutamine and D-glutamate metabolism > butanoate metabolism > arginine and proline metabolism > pantothenate and CoA biosynthesis > biosynthesis of unsaturated fatty acids > glycine, serine and threonine metabolism > cyanoamino acid metabolism > glutathione metabolism > galactose metabolism (Figure 2(a)). Integrative analysis of metabolites in the enriched pathways is described in Figure 2(b). Red and blue indicated increased and decreased metabolites, respectively. Of particular interest were the metabolites of the TCA cycle. Out of six identified TCA cycle metabolites, five were increased in abundance and one, pyruvic acid, was decreased (Figure 2(b)). Moreover, the first enriched pathway (alanine, aspartate, and glutamate metabolism) contains almost metabolites detected in the TCA cycle except for alanine, glutamine, and asparagine. In addition, the three metabolites are closely related to the TCA cycle, fuel or/and the production of the TCA cycle. These results indicate that the increased TCA cycle is a characteristic metabolic feature of the malate-reprogramming metabolome.

### Crucial biomarkers in the malate-reprogramming metabolome

**Figure 3.**
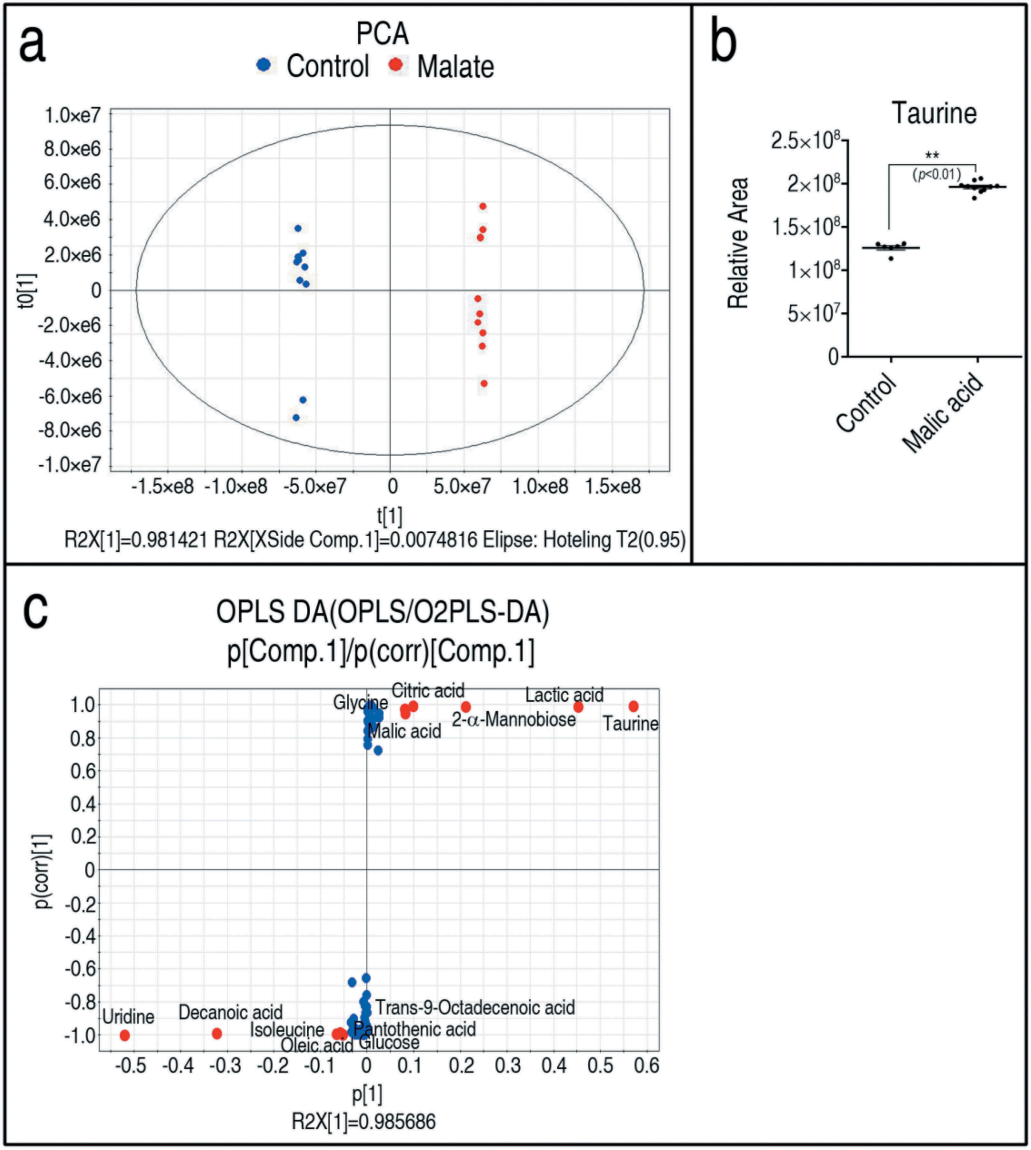
Identiﬁcation of crucial metabolites. (a) PCA analysis of malic acid and control groups according to the treatments set. Each dot represents the technical replicate analysis of samples in the plot. T [1] and t0 [1] used in this plot explain 98.14% of the total variance which allows conﬁdent interpretation of the variation. (b) S-plot generates from OPLS-DA (R2X = 0.986 R2Y = 0.999, Q2 = 0.997). Predictive component p [1] and correlation p(corr) [1] differentiate malic acid from control. Dot represents metabolites and candidate biomarkers are highlighted in red. (c) Scatter plot of taurine, which comes from data 1a. Results (c) are displayed as mean ± SEM, and significant differences are identified (**p < 0.01) as determined by two-tailed Student’s *t*-test.

To explore the most crucial metabolites differentiating the reprogramming group from the control, orthogonal partial least square discriminant analysis (OPLS-DA) was conducted to recognize the sample pattern. The two groups were distributed in two quarters (R2X = 0.986 R2Y = 0.999, Q2 = 0.997). Component p [1] separated the reprogramming group from the control group, and component p [2] differentiated variants within groups (Figure 3(a)). Discriminating variables were shown with an S-plot when cutoff values were set as greater or equal to 0.05 and 0.5 for the absolute value of covariance p and correlation p(corr), respectively. Thus, 13 metabolites were identified as biomarkers, where the abundance of 6 metabolites (taurine, lactic acid, mannobiose, citric acid, glycine, and malic acid) was increased, and the abundance of 7 metabolites (uridine, decanoic acid, isoleucine, oleic acid, glucose, pantothenic acid, and trans-9-octadecenoic acid) was decreased (Figure 3(b)). Among the 6 increased metabolites, taurine had the highest absolute value of covariance and thereby was identified as the most crucial biomarker for further functional study. The relative area of taurine between the reprogramming group and the control is shown in Figure 3(c).

### iPath analysis

**Figure 4.**
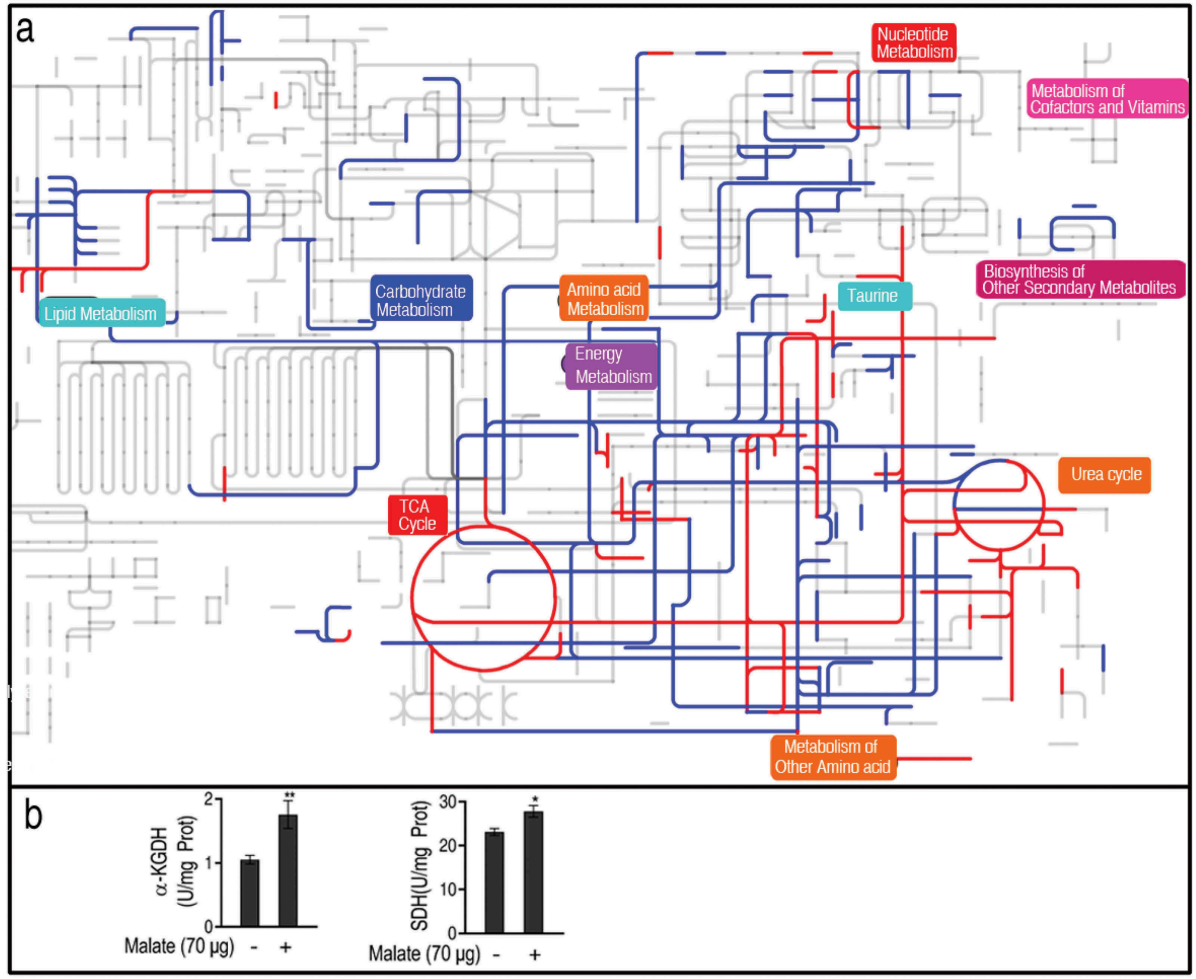
Comparative metabolic pathway analysis. (a) Analysis of the metabolic profiles resulting from *D. rerio* injected by 70 μg malic acid provides a better insight into the effects of 90 significant metabolites (*p *< 0.01). Based on the KEGG compound (http://www.kegg.jp/kegg/compound/), metabolic network pathways in *D. rerio* are further analyzed with iPath2.0 (http://pathways.embl.de/iPath2.cgi). Red and blue lines represent increase and decrease in the malic acid group, respectively. (b) The activity of a-ketoglutaric dehydrogenase (KGDH) and succinate dehydrogenase (SDH). Twenty zebrafish spleens were collected in each group. Five were pooled as a sample, yielding four biological repeats for analysis of enzyme activity. Samples were collected in 24 h after the injection of malate for 3 days. Results (b) are displayed as mean ± SEM, and significant differences are identified (*p < 0.05, **p < 0.01) as determined by two-tailed Student’s *t*-test.

A comparative metabolic pathway analysis between the reprogramming group and the control was carried out by iPath 2.0 [29]. The resulting global overview map provided a better insight into the effects of exogenous malate on the metabolism of the fish, where red and blue lines represented increased and decreased pathways in the reprogramming group, respectively. The TCA cycle, together with the pathways mediating the TCA cycle to taurine biosynthesis, was increased (Figure 4(a)), implying the importance of the activation of the TCA cycle and taurine biosynthesis. Thus, the activity of enzymes in the TCA cycle was measured. The activity of α-ketoglutarate dehydrogenase (KGDH) was increased by 66.8% and succinate dehydrogenase (SDH) was increased by 20.0% (Figure 4(b)), which supports the conclusion that malate promotes the TCA cycle.

### Malate promotes taurine biosynthesis and taurine potentiates zebrafish against V. alginolyticus infection

**Figure 5.**
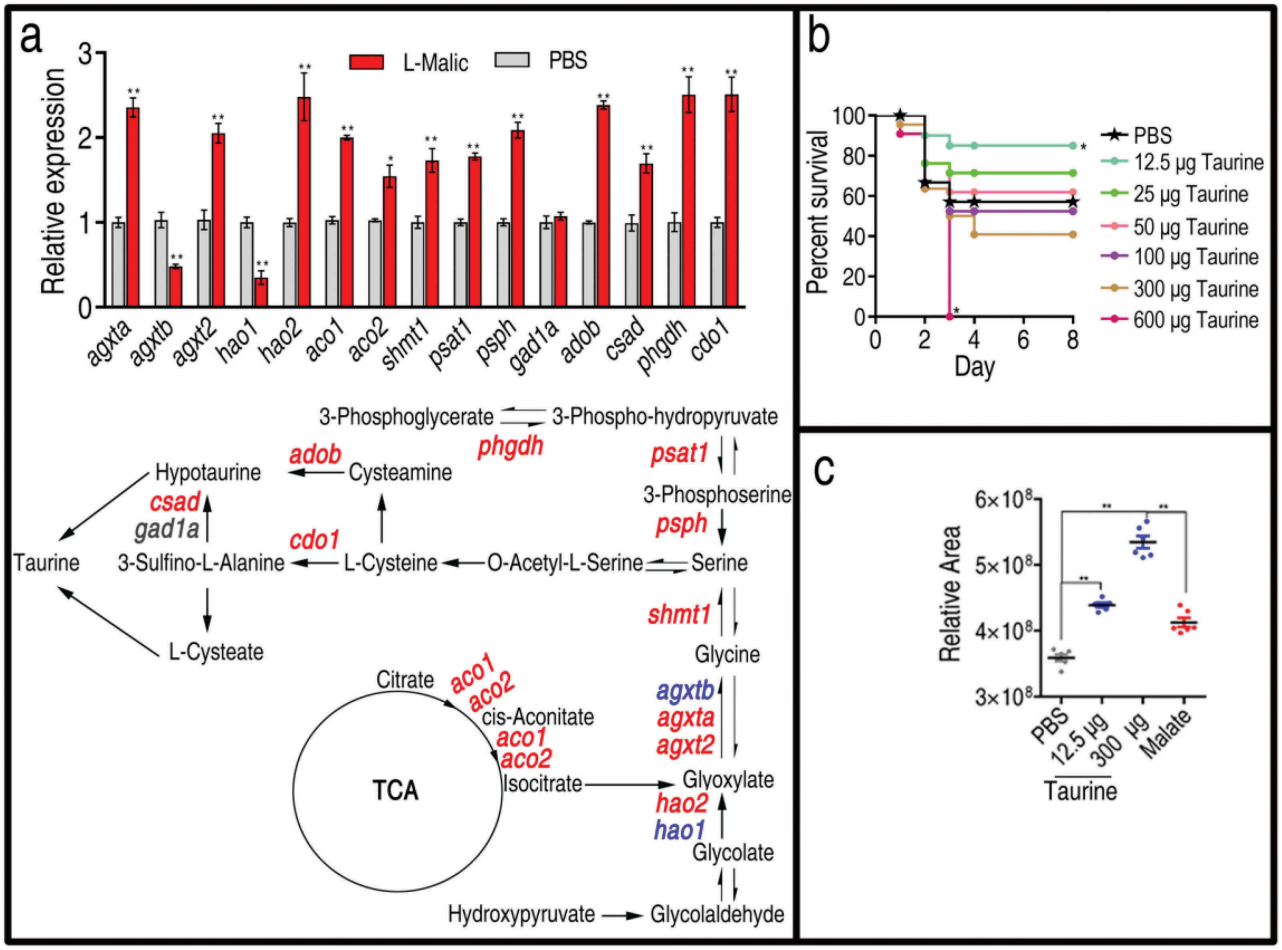
Taurine biosynthesis pathway and ability against bacterial infection. (a) qRT-PCR for expression of taurine biosynthesis pathway genes. Upper, relative gene expression; Lower, shown in the metabolic pathway. Twenty-five zebrafish spleens were collected in each group. Five were pooled as a sample, yielding five biological repeats for analysis of gene expression. Samples were collected in 24 h after the injection of malate for 3 days. qRT-PCR was based on the key genes of taurine biosynthesis pathways in *D. rerio*. Red represents increase, blue represents decrease, and black represents unchanged in the malate-reprogramming group. (b) Percent survival of zebrafish in the presence of the indicated doses of taurine, 21 zebrafish each group. (c) Relative concentrations of taurine in the presence of PBS, 12.5 μg of taurine, 300 μg of taurine, and 70 μg of malate, five zebrafish each group. Taurine is measured by GC-MS analysis. Results are displayed as mean ± SEM, and significant differences are identified (*p < 0.05, **p < 0.01) as determined by two-tailed Student’s *t*-test (a and c) and Log-rank (Mantel-Cox) test, Gehan-Breslow-Wilcoxon test, and Tarone-Ware test (b).

The above results indicate that the elevated TCA cycle and taurine represent the most characteristic features of the reprogramming group. Therefore, we speculate that exogenous malate promotes the TCA cycle and, in turn, the TCA cycle provides isocitrate for the transformation of taurine via serine. To test this, qRT-PCR was used to quantify the expression of genes in the transformation pathway. Among the 16 genes detected, the expression of 13 genes was increased, that of 1 gene was unaffected, and that of 2 genes was decreased (Figure 5(a)). These results indicate that the elevated taurine was attributed to the metabolic transformation from the activated TCA cycle. Then, we tested whether taurine provides protection against infection caused by *V. alginolyticus* in zebrafish as malate did [28]. Thus, 12.5 μg, 25 μg, 50 μg, 100 μg, 300 μg, or 600 μg taurine was injected into zebrafish followed by *V. alginolyticus* challenge. Surprisingly, zebrafish survival was inversely correlated with the taurine dosages. Specifically, 57.14%, 85.71%, 71.43%, 61.43%, 52.38%, 38.1%, and 0% survival was observed at 0 μg,12.5 μg, 25 μg, 50 μg, 100 μg, 300 μg, and 600 μg taurine, respectively (Figure 5(b)). These results indicate that a low concentration of taurine provides immune protection for zebrafish against the bacterial infection, as malate did. However, a high concentration of taurine showed the opposite effect. Consistently, 12.5 μg taurine elevated that taurine level (22.3%) similar to what 70 μg malate (15.0%) did, whereas 300 μg taurine elevated the taurine level by approximately 48.9%, which were detected in humoral fluid by GC-MS (Figure 5(c)). These results indicate that low and high endogenous taurine levels may promote zebrafish survival and causes zebrafish death, respectively.

### Malate and taurine stimulate innate immunity similarly

**Figure 6.**
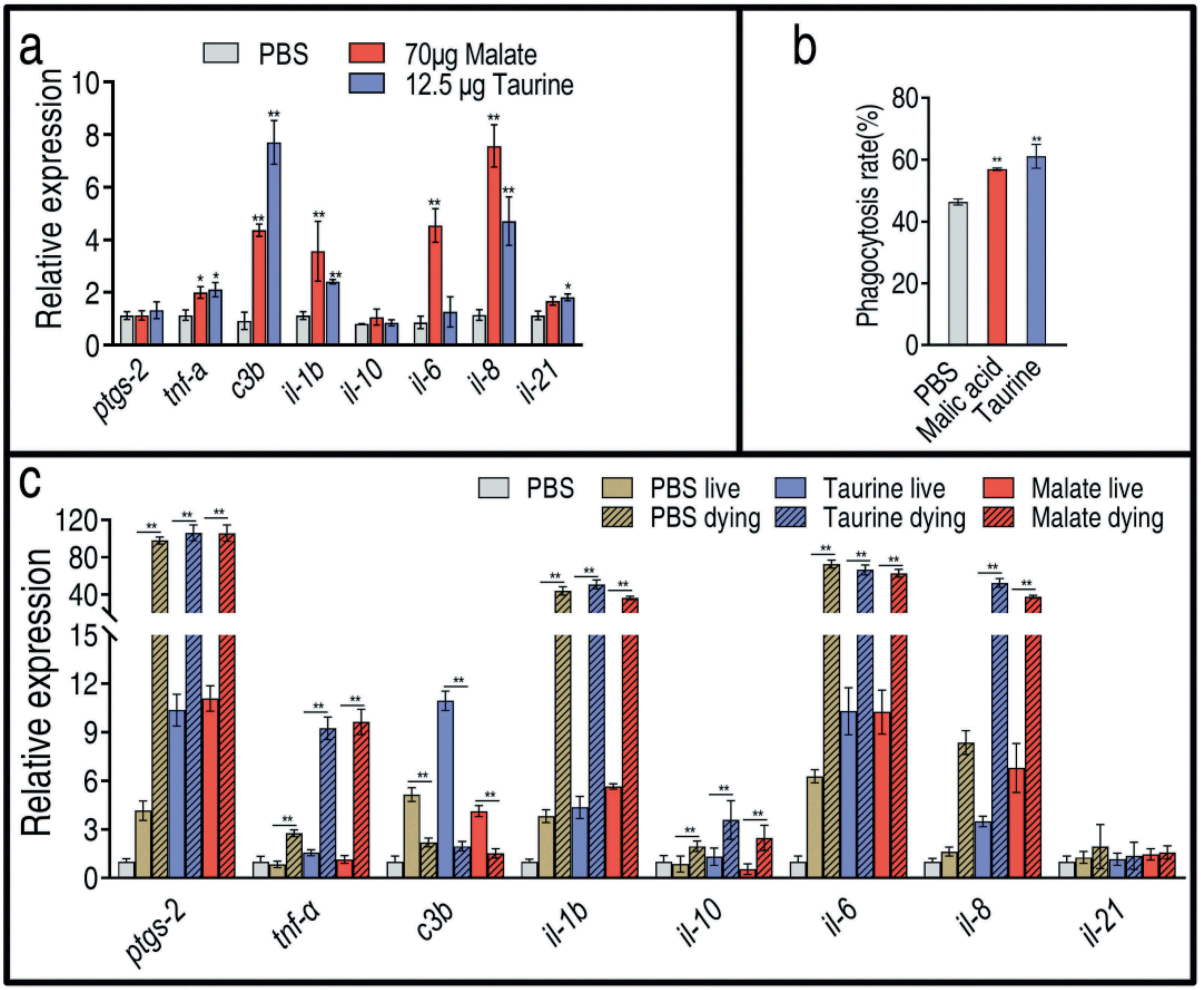
Expression of innate immunity genes in the presence of malate or taurine or/and bacterial infection. (a) Expression in the absence or presence of 70 μg malate or 12.5 μg taurine. Twenty-five zebrafish spleens were collected in each group. Five were pooled as a sample, yielding five biological repeats for analysis of gene expression. Samples were collected in 24 h after the injection of malate or taurine for 3 days. (b) Phagocytosis in the absence or presence of 70 μg malate or 12.5 μg taurine. Macrophages were separated from head kidney of Nile tilapia and incubated with 20 mM malate or taurine. Then, 1:100 bacterial cells were added. Three biological repeats were performed. (c) qRT-PCR for expression of innate immune genes post bacterial infection. Twenty-five zebrafish spleens were collected in each group. Five were pooled as a sample, yielding five biological repeats for analysis of gene expression. Zebrafish were treated with PBS, malate, or taurine for 3 days and then challenged by bacteria. Samples were collected at 30 h post the bacterial challenge. They included PBS group, only PBS injection without bacterial challenge; PBS live group, survival after PBS injection and bacterial challenge; PBS dying, dying after PBS injection and bacterial challenge; Taurine live, survival after taurine injection and bacterial challenge; Taurine dying, dying after taurine injection and bacterial challenge; Malate live, survival after malate injection and bacterial challenge; Malate dying, dying after malate injection and bacterial challenge. Results are displayed as mean ± SEM, and significant differences are identified (*p < 0.05, **p < 0.01) as determined by two-tailed Student’s *t*-test.

The above results indicate that exogenous malate promotes taurine production and, in turn, taurine potentiates zebrafish to fight against *V. alginolyticus*, suggesting that malate exhibits potential via taurine, which may have a similar capability to stimulate an innate immune response in the same way that malate did. To demonstrate this, qRT-PCR was used to quantify expression of eight innate immunity genes in the presence of exogenous malate or taurine. The expression levels of all genes were similar between the two treatments. Specifically, the expression of *il-6, il-1b, il-8, tnf-a*, and *c3b* was elevated, and the expression of *il-6* and *il-1b* was higher in the malate group than in the taurine group. In addition, no expression difference in *il-10, il-21*, and *ptgs-2* (cyclooxygenase-2) was found between the two groups (Figure 6(a)). These results indicate that cell phagocytic capacity is activated by the two metabolites, which was further demonstrated by the elevated phagocytosis of tilapia primary phagocytes in response to *V. alginolyticus* (Figure 6(b)). When zebrafish were infected with *V. alginolyticus*, living and dying zebrafish exhibited differential innate immune responses. Specifically, higher expression levels of *ptgs-2, tnfa, c3b, il-1b, il-*6, and *il-*8 and a lower expression level of *c3b* were detected in the dying fish but not in the living fish (Figure 6(c)). Thus, 12.5 μg taurine or 70 μg malate alleviated the high expression of innate immune genes but promoted the low expression of innate immune genes, and thereby elevated the survival of zebrafish (Figure 5(b)). These results indicate that taurine regulates the innate immune response in a manner similar to that of malate.

### Malate and taurine influence inducible nitric oxide synthase, ROS, and glutathione peroxidase

**Figure 7.**
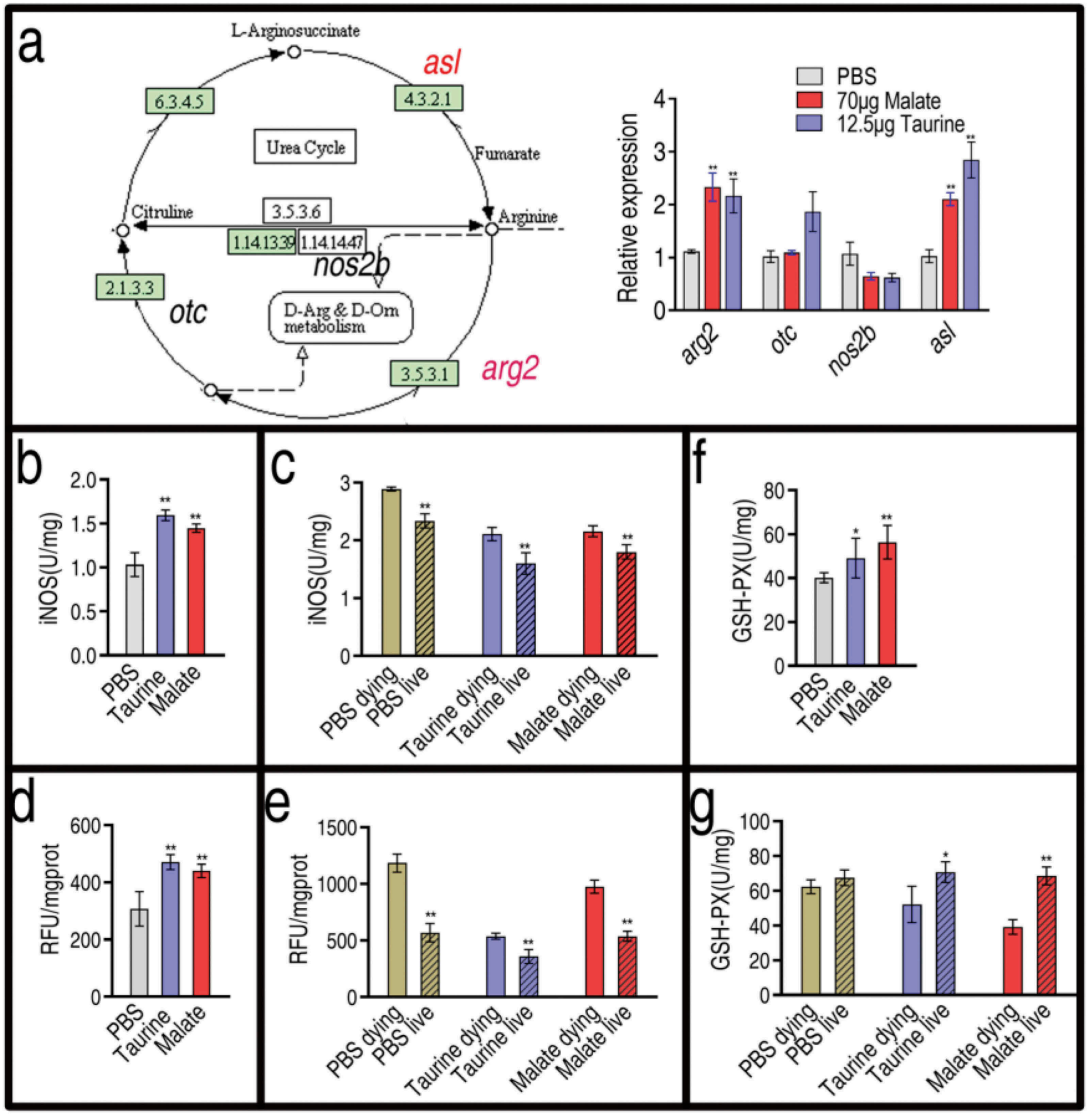
Effect of malate and taurine on gene expression of NO biosynthesis. (a) Expression of NO biosynthesis genes in the absence or presence of 70 μg malate or 12.5 μg taurine. Left, shown in the metabolic pathway; Right, relative gene expression. (b) The activity of iNOS in the absence or presence of 70 μg malate or 12.5 μg taurine. (c) The activity of iNOS in samples as Figure 6c post bacterial challenge. (d) ROS in the absence or presence of 70 μg malate or 12.5 μg taurine. (e) ROS in samples as Figure 6c post bacterial challenge. (f) GSH-PX in the absence or presence of 70 μg malate or 12.5 μg taurine. (g) GSH-PX in samples as Fig 6 C post bacterial challenge. (a, b, d, and f) Twenty zebrafish spleens were collected in each group. Five were pooled as a sample, yielding four biological repeats for analysis of gene expression. Samples were collected in 24 h after the injection of malate or taurine for 3 days. (c, e, and g) Twenty zebrafish spleens were collected in each group. Five were pooled as a sample, yielding four biological repeats for analysis of gene expression. Zebrafish were treated with PBS, malate, or taurine for 3 days and then challenged by bacteria. Samples were collected at 30 h post the bacterial challenge. They included PBS group, only PBS injection without bacterial challenge; PBS live group, survival after PBS injection and bacterial challenge; PBS dying, dying after PBS injection and bacterial challenge; Taurine live, survival after taurine injection and bacterial challenge; Taurine dying, dying after taurine injection and bacterial challenge; Malate live, survival after malate injection and bacterial challenge; Malate dying, dying after malate injection and bacterial challenge. Results are displayed as mean ± SEM, and significant differences are identified (*p < 0.05; **p < 0.01) as determined by two-tailed Student’s *t*-test.

Reports indicate that taurine attenuates inflammation via decreasing inducible nitric oxide synthase (iNOS) and ROS in rats and mammary epithelial cells [30–32]. Thus, we investigated the effect of malate and taurine on nitric oxide (NO) and ROS production in fish. Both exogenous metabolites regulated the expression of genes related to NO generation in a similar way. Among the four genes detected, the expression of three genes, *arg*2, *otc* (except for malate), and *asl*, showed increased expression, and the expression of one gene, *nos2*b, was unaffected (Figure 7(a)). In addition, activity of iNOS was quantified. Malate and taurine increased the activity of iNOS (Figure 7(b)). Further results showed that the activity of iNOS was higher in the dying fish than the living fish after bacterial infection, and the activity of both groups was higher than that of the control group without bacterial infection. However, pre-treatment of exogenous taurine or malate followed by bacterial challenge led to similar activity of iNOS between the control without bacterial infection and the live fish, which was lower than that of the dying group (Figure 7(c)). Furthermore, we detected the ROS level in the same samples. Similarly, ROS was elevated in the presence of exogenous malate or taurine (Figure 7(d)), and a higher ROS was measured after bacterial infection. The ROS level returned to normal in the live fish after bacterial infection when the fish were pre-treated with taurine or malate (Figure 7(e)). The glutathione metabolic pathway was also enhanced, as shown in Figure 2(b). Thus, the activity of glutathione peroxidase (GSH-PX) was measured. Consistently, fish pre-treated with taurine or malate exhibited higher activity of the enzyme (Figure 7(f)). When infected with *V. alginolyticus*, dying fish exhibited lower activity of GSH-PX than did the living fish (Figure 7(g)). These results indicate that exogenous taurine modulates NO and ROS to fight bacterial infection.

## Discussion

Bacterial disease outbreak is a significant constraint to the development of the aquaculture sector. Besides vaccination and antibiotic approaches [8,10,33,34], alternative strategies are being developed to control bacterial diseases [20,35,36]. These include the specific killing of pathogenic bacteria (bacteriophages and inhibition of chromosome II replication in vibrios), growth inhibition (short-chain fatty acids and polyhydroxyalkanoates), inhibition of virulence gene expression (interfering with the regulation of virulence factor expression and specifically inhibiting a certain virulence factor), and reprogramming of the antibiotic-resistant metabolome (reverting bacterial antibiotic resistance to uptake antibiotics) [35,37,38]. Recently, we have developed reprogramming metabolomics to modulate the host metabolome against bacterial infection without the use of antibiotics [19,20], where malate is identified as a crucial reprogramming molecule [28]. Malate boosts the TCA cycle to enhance the survival of zebrafish to *V. alginolyticus* infection [28] but the underlying mechanism is unknown. The present study explores the mechanisms by which malate reprograms the fish’s metabolome to fight against *V. alginolyticus* infection. Our results indicate that malate enhances the survival of zebrafish against infection in the same manner as taurine.

Taurine is a metabolite that is widely distributed in animal tissues, including fish tissues [39]. Taurine is derived from cysteine and transformed by serine from the isocitrate of the TCA cycle [40]. The present study used the reprogramming metabolomics approach and iPath analysis to demonstrate TCA cycle activation and the malate-induced elevation of taurine by exogenous malate. Furthermore, the elevated expression of genes in the taurine biosynthesis pathway is documented. Thus, exogenous malate promotes the biosynthesis of taurine.

Furthermore, we investigated whether exogenous taurine protects zebrafish from infection in the same way that malate did. Reports indicate that taurine is used for the treatment of various superficial infections and chronic inflammation. In aquaculture, dietary taurine improves growth, regulates immunity, and enhances the antioxidant capacity in crab (*Eriocheir sinensis*) [41]. Taurine supplementation affects the metabolome, improves energy utilization and amino acid uptake, promotes protein, lipid, and purine synthesis, and accelerates growth in grouper (*Epinephelus coioides*) [42]. However, Kortner et al. indicated that dietary supplementation with 0.4% taurine in salmon does not relieve gut enteritis, which is the first pathogenic stage of vibriosis with enteritis [43]. In addition, the depletion of taurine is found in the gills of female abalones infected with *V. parahemolyticus* [44] and in natural skin ulceration syndrome-diseased sea cucumber [45]. In mammals, taurine regulates the inflammatory response during bacterial infection by potentiating the phagocytic activity of macrophages, affecting the signaling pathways, and improving the antioxidant ability of cells [30,46]. Taurine-conjugated bile acids are identified as inhibitors of biofilm formation against both *Vibrio cholerae* and *Pseudomonas aeruginosa* [47]. Taurine complementation attenuates pro-inflammatory response in mammary epithelial cells after *Streptococcus uberis* challenge [48] and in a bovine mammary epithelial cell (MEC) line (MAC-T) or a mouse mammary epithelial cell line (EpH4-Ev) infected with *Escherichia coli* and *Staphylococcus aureus* [30]. In addition, exogenous taurine also promotes the anti-infective ability against viruses and fungi [49,50].

The present study showed that 70 μg exogenous malate elevated the taurine level, which is similar to an injection of 12.5 μg taurine. Both 12.5 μg exogenous taurine and 70 μg exogenous malate showed similar effects in increasing survival of zebrafish infected with *V. alginolyticus*. These results support the conclusion that taurine biosynthesis is crucial to fight against the bacterial infection in the malate-reprogramming metabolome. In addition, the present study found that a high concentration of exogenous taurine causes increased death. Indeed, injection with 300 μg taurine increased the taurine level by approximately 2.19–3.26 fold compared with the level of increase with 12.5 μg exogenous taurine and 70 μg exogenous malate. Thus, the action of taurine in fighting bacterial infection is related to its dosage in zebrafish, which may be related to why the dietary supplementation with 0.4% taurine does not relieve gut enteritis in salmon [43].

The immune system is essential for the control and elimination of infections. Thus, further investigation on the relationship between taurine and innate immune responses stimulated by malate was carried out. IL-1β, IL-6, IL-8, IL-10, and IL-21 are cytokines that mediate different kinds of immune responses (34). Specifically, IL-1β is a pro-inflammatory cytokine produced in a variety of cells, including monocytes, tissue macrophages, keratinocytes, and other epithelial cells [51]. IL-6 functions in inflammation and the maturation of ** [52] B cells through activation of Janus kinases (JAK) and signal transducers and activators of transcription (STAT1 and STAT3) (54). IL-8 is a pro-inflammatory and growth-promoting factor [50,53]. IL-10 is a type II cytokine with numerous immunosuppressive and anti-inflammatory capacities, playing a role in the inhibition of antigen-presenting cells, including dendritic cells, monocytes, and macrophages [50]. IL-21 is a type I cytokine produced by activated T cells that promotes cytokine production in monocytes [54]. TNF-α is a potent pro-inflammatory cytokine exerting pleiotropic effects on various cell types [55]. C3b is a key component of the complement system. COX-2, an inducible cyclooxygenase, is produced in abundance by activated macrophages and other cells at the site of inflammation, playing a key role as a pro-inflammatory molecule in fish [56,57]. The present study showed that malate and taurine promote the expression of genes encoding IL-1β, IL-6, IL-8, and C3, while the higher expression of genes encoding IL-1β, IL-6, IL-8, and Cox-2 and lower expression of gene encoding C3 were detected in the dying fish but not in the living fish. Interestingly, taurine and malate alleviated the expression of Cox-2, IL-1β, IL-6, IL-8, and elevated the expression of C3 after bacterial infection. This alleviation and elevation increased fish survival.

The present study further explored whether taurine and malate influence NO and ROS levels and whether the influence was related to anti-infection in zebrafish. Taurine promoted NO biosynthesis and iNOS and ROS levels in the same way that malate did. The higher iNOS and ROS levels in the dying fish rather than the living fish indicate that excessive responses of iNOS and ROS are associated with the lower survival caused by a bacterial infection. Taurine weakened the responses and thereby elevated the survival. Importantly, the ROS level was related to the action of GSH-PX. These data are consistent with previous reports that taurine ameliorated the elevated iNOS and ROS caused by bacterial infection in rats and mammary epithelial cells [30–32]. Thus, that low concentration of taurine leads to the increased survival of zebrafish infected with *V. alginolyticus* is partly attributed to adjustments to inflammation-related innate immunity, NO, and ROS levels. Logically, the data showing that high concentrations of taurine cause a reduction in survival suggest the consequence of the excessive inhibition of these responses. This finding is useful for establishing a reasonable level of taurine use in aquaculture.

Our results show that malate supplementation will result in increased survival, but a range of additional studies, including full-scale field studies will need to be carried out before the approach is adopted by farmers.

In summary, the present study demonstrates that malate potentiates zebrafish against infection caused by *V. alginolyticus* via taurine. This is done through regulating the innate immune response and inhibiting NO and ROS. Thus, our study unravels the mechanisms by which the malate-reprogramming metabolome fights against infection by promoting taurine biosynthesis. Furthermore, the present study indicates that a high dose of taurine is not suitable for fighting against bacterial infection in aquaculture.

## Materials and methods

### Animals and bacterial strain

Zebrafish AB lines, *Danio rerio*, about 3 months (body length: 2.5 ± 0.2 cm, body weight: 0.26 ± 0.04 g) were purchased from Shaoping Corp. Guangzhou Fangcun Huadiwan Flower Bird Fish & Insect Market, Guangzhou, China. Nile tilapia, *Oreochromis niloticus*, about 500 ± 10 g, were obtained from Guangdong Tilapia Breeding Farm (Guangzhou, China). These animals were acclimated in 8 water tank (80 cm × 75 cm × 90 cm) equipped with Closed Recirculating Aquaculture Systems, and the maintaining physico-chemical parameters were: water temperature: 27–29°C, dissolved oxygen: 6–7 mg/L, carbon dioxide content: <10 mg/L, pH value: 7.0–7.5, nitrogen content: 1–2 mg/L, and nitrite content: 0.1–0.3 mg/L. They were fed with fish food (Jinfeng pellets, 38% crude protein, 6% crude fat, and 16% crude ash related to wet matter, 7% crude fiber and 8% moisture, based on NRC recommendations, at a ratio of 3% of body weight per day) twice a day for 2 weeks on a 12 h/12 h rhythm of light and darkness photoperiod before experimental manipulation. These tanks were cleaned twice a day by siphoning up the food debris and feces [14]. These animals were demonstrated to be free from *Vibrio spp*. infection by bacteriology and *gyrB*-specific primer PCR before using in subsequent experiments. The rearing and treatment of the experimental fish were approved by Sun Yat-sen University. *V. alginolyticus* V12G01 was from the collection of our laboratory. A single colony was cultured in Luria-Bertani (LB) medium (1% w/v peptone, 0.5% w/v yeast extracts, 1% w/v NaCl, pH7.4) at 30°C overnight in a shaker as seed. The overnight cultures were diluted 1:100 in fresh LB medium, cultured at 30°C and grown to an OD_600_ of 1.0.

### Supplementation of exogenous metabolites and bacterial challenge

Exogenous metabolites were supplemented as previously described [28]. Zebrafish were intraperitoneally injected by 10 μL with a dose of 70 μg malate or 12.5 μg taurine dissolved in phosphate buffer saline (PBS) for 3 days, once a day, as a tested group and by 10 μL PBS buffer as a control. Humoral fluid was collected for GC-MS analysis and spleen samples were collected for qRT-PCR, enzyme activity, iNOS, and ROS detection after 24 h. For bacterial challenge, a single colony was cultured in Luria-Bertani (LB) medium (1% w/v peptone, 0.5% w/v yeast extracts, 1% w/v NaCl, pH7.4) at 30°C overnight in a shaker as seed. The overnight cultures were diluted 1:100 in fresh LB medium, cultured at 30°C and grown to an OD_600_ of 1.0. Then, the bacteria were collected by centrifugation, washed and resuspended to OD_600_ 0.2 by sterile saline solution. To determine the half-lethal dose (LD50), *Danio rerio* were infected with three different doses of bacteria through intramuscular injection (by inserting the needle into the left tail muscle for 2–3 mm from the body in horizontal), including 2 × 10^5^, 4 × 10^5^ and 6 × 10^5^ CFU/fish, with *Danio rerio* receiving each dose. The LD50 was 2 × 10^5^ CFU/fish. Zebrafish were intraperitoneally injected by 10 μL with a dose of 70 μg malate, 12.5 μg, 25 μg, 50 μg, 100 μg, 300 μg, or 600 μg taurine dissolved in PBS buffer for 3 days, once a day, as a tested group and by 10 μL PBS buffer as a control. Then, each fish was challenged by 2 × 10^5^ CFU/fish at 24 h later. Percent survival was obtained by signs of infection and mortality recorded twice a day for 8 days. The infected zebrafish showed signs of infection at 24 h post infection and most died within 48 h. Therefore, samples were collected in 30 h for analysis of qRT-PCR, iNOS, and ROS of live and dying zebrafish.

### Sample preparation for GC-MS analysis

Zebrafish humoral fluid was collected as previously described with some modifications [28]. In brief, zebrafish were treated with 70 μg malate/10 μL PBS and with 10 μL PBS as a reprogramming group and a control, respectively, five fish each group. These treated animals were rinsed with a distilled saline solution and then wiped thoroughly with sterilized gauze. These animals were cut into five pieces on ice and then weighted. The appropriate volume of saline (100 μL/100 mg) was added according to the weight. After centrifugation at 3000 × g, 4°C, 100 μL fluid was isolated for the further study of metabolites. Metabolites were extracted with 0.2 mL cold methanol (Sigma). Then, 10 µL ribitol (0.1 mg per mL) was added into each sample tube as an internal quantitative standard. After centrifugation at 12,000 × g for 10 min, and transferred to new 1.5 mL centrifuge tubes. Then, the supernatants were concentrated in a rotary vacuum centrifuge device (LABCONCO). The dried polar extracts were used for metabolite derivatization of GC-MS analysis, the dried residue was dissolved in 100 µL 20 mg per mL methoxyamine pyridine solution and incubated for 120 min at 37°C in an incubator shaker. Finally, the mixture was treated with 100 µL N,O-Bis(trimethylsilyl)trifluoroacetamide (TMSTFA) with 1% trimethylchlorosilane (TMCS) and incubated for 30 min at 37 ° C. Every experiment was repeated by five biological replicates.

### GC-MS detection

The derivatized sample of 1 μL was injected into an HP-5 MS column (Agilent Technologies, 30 m × 250 μm × i.d. 0.25 μm) using splitless injection and analysis was carried out by Agilent 7890A GC equipped with an Agilent 5975 C VL MSD detector (Agilent Technologies). The initial temperature of the GC oven was held at 85°C for 5 min followed by an increase to 280°C at a rate of 15°C per min holding for 5 min and then increased to 310°C at a rate of 20°C per min. The solvent delay time was set as 5 min before data collection. High purity helium (purity not less than 99.999%) was used as a carrier gas and flow was kept constant at 1 mL per min. The scanning mode is full scan (SCAN), and MS was operated in a range of 50–600 m/z.

### Spectra processing for GC-MS

The deconvolution and calibration of the acquired mass spectra were performed with AMDIS (Agilent OpenLAB CDS ChemiStation C.01.01). To avoid false positives, peaks with a signal-to-noise ratio (S/N) lower than 30 were excluded [58]. Additionally, the artifact peaks were removed through comparison with the blank samples. Metabolites were identified by retrieving their mass spectra in the NIST 2011 (National Institute of Standards and Technology, USA) library and GMD 2011 (Golm Metabolome Database, Germany) according to the following criteria: match value ≥ 750, reverse match value ≥ 800, and a probability ≥ 60% [59]. The relative peak area value of adonitol was taken as the internal standard to calculate the metabolite abundance. The zeroes or missing values were assessed by singular value decomposition (SVD) method to impute the missing values. Variables were removed for threshold 50%, and missing values were replaced with an average value. Then, these data were improved by interquartile range (IQR) filtering. The none option was used for filtration, which applied to the number of variables of samples with less than 5000. The combined normalization processing consisted of the following options: quantile normalization row-wise procedures and pareto scaling (mean-centered and divided by the square root of standard deviation of each variable). The data array file can be used for the subsequent multivariate statistical analysis.

### Bioinformatics analyses

Data transformations and manipulations were done using Excel. The differences in the metabolite contents between the two groups were compared using the Mann–Whitney U test (α = 0.05) with the SPSS 23.0 (IBM, USA). Prior to analysis, sets of metabolites data subtracted the median metabolites and were scaled by the quartile range in the sample. A multivariate statistical analysis of the metabolomic data was further performed using the MetaboAnalyst online website (www.metaboanalyst.ca) [60,61]. Z-score analysis scaled each metabolite according to a reference distribution and calculated based on the mean and standard deviation of reference sets a control. Z-score formula was shown as followed, $Z=\frac{x_{ij}-AVG_{i}}{SD_{i}}$, x_ij_ represented metabolites’ peak area, AVG_i_ represented average of control group, SD_i_ represented standard deviation of control group. Using gplots package of R project (R i386 3.4.3, www.r-project.org), a hierarchical cluster analysis (HCA) was first performed using the distance matrix calculated with the Euclidean method. Using SIMCA-P 13.0 (Umetrics, Sweden), the principal component analysis (PCA) and orthogonal partial least squares-discriminant analysis (OPLS-DA) was then conducted to investigate the relationships among the test samples. Based on the OPLS-DA s-plot analysis, the compounds with a variable weight value of p [1] and p(corr) [1] were greater than 5 and 0.5 or were lower than −5 and −0.5, respectively, which were filtered and shown in a scatter plot. For the pathway enrichment analysis, the metabolic pathways involving the metabolites that showed differences between the test groups were identified using the MetaboAnalyst online website (www.metaboanalyst.ca) [61,62]. The -log(p) value ** [63] and a value reflecting the impact of each metabolic pathway were calculated using a hypergeometric test, and the metabolic pathways with *p* < 0.05 were retained. Prism v5.01 (GraphPad, La Jolla, CA, USA) was used to draw the histogram the scatter plot. Comparative metabolic pathway analysis between the two groups was performed using iPath 3.0 (https://pathways.embl.de/)

### Measurement of activity of α-ketoglutaric dehydrogenase, succinate dehydrogenase, and glutathione peroxidase

Measurement of activity of α-ketoglutaric dehydrogenase (KGDH) and succinate dehydrogenase (SDH) was performed as previously described [28]. In brief, zebrafish were randomly divided into two groups treated with and without 70 μg malate, 20 each. Five spleens were pooled as a biological sample and four biological repeats were carried out. Supernatants from these spleen homogenate were obtained, which contained 400 μg or 200 μg total proteins and were transferred to the KGDH reaction mix (0.5 mM MTT, 1 mM MgCl2, 6.5 mM PMS, 0.2 mM TPP, 50 mM PBS, and 2 mM sodium α-ketoglutaric for KGDH) or SDH reaction mix (0.5 mM MTT, 13 mM PMS, 5 mM succinate, 50 mM PBS), respectively, to a final volume of 200 mL in 96-well plate. Subsequently, the plate was incubated at 37°C for 30 min for KGDH and 10 min for SDH and then measured at 566 nm for colorimetric reading. Measurement of glutathione peroxidase (GSH-PX) activity was used as a commercial ELISA kit for fish (KT57683, MSKBIO Ltd., Wuhan, China). The above 10 μL sample was added into 40 μL diluent in each well, mixed, and incubated at 28°C for 30 min. After washing for 5 times, 50 μL enzyme-linked reagent was added and incubated at 28°C for 30 min. The same washing was performed and then 50 μL coloration buffer A and B was added and incubated at 28°C in dark for 15 min. Finally, 50 μL termination buffer was added to turn off the coloration reaction and the absorption value at 450 nm in 15 min after termination using a microplate spectrophotometer (Epoch2, BioTek Instruments Inc., USA).

### Quantitative real-time PCR (qRT-PCR)

qRT-PCR was performed as previously described [62]. Zebrafish were randomly divided into seven groups, 25 each. They included a PBS control without bacterial infection and six groups with bacterial infection (PBS-teated dying group, PBS-treated survival group, malate-treated dying group, malate-treated survival group, taurine-treated dying group, taurine-treated survival group). Spleens from five fish were pooled as a biological sample for RNA extraction, five biological repeats each group. Total RNA was extracted using TRIZOL reagent (Ambion Life Technologies) according to the manufacturer’s protocol. RT-PCR was carried out on 1 μg total RNA with PrimeScript RT reagent kit with gDNA Eraser (Takara, Japan) according to manufacturer’s instructions. Two technical repeats were carried out for each biological sample. qRT-PCR was performed on a LightCycler 480 system (Roche, Germany) and SYBRPremix Ex TaqTM II (Takara, Japan). The cycling parameters were listed as follows: 95°C for 30 s to activate the polymerase; 40 cycles of 95°C for 10 s; 60°C for 30 s; Fluorescence measurements were performed at 70°C for 1 s during each cycle. Cycling was terminated at 95°C with a calefactive velocity of 5°C per second and a melting curve was obtained. Gene-specific primers used for qRT-PCR are shown in **Supplementary Tab. 1**. The relative expression of each immune-related gene was calculated by 2^−ΔΔCt^ method (66) using β-actin as a reference gene.

### iNOS detection

Inducible Nitric Oxide Synthase (iNOS) Assay Kit (A014-1) was purchased from the Nanjing Jiancheng Bioengineering Institute, China. Zebrafish were randomly divided into seven groups, 20 each. They included a phosphate buffer saline (PBS) control without bacterial infection and six groups with bacterial infection (PBS-treated dying group, PBS-treated survival group, malate-treated dying group, malate-treated survival group, taurine-treated dying group, taurine-treated survival group). Spleen samples were collected and homogenated with phosphate buffer (pH = 7.4) at 1:15 (w/v). Supernatants were collected by centrifugation. Protein concentration was determined using the BCA Protein Assay Kit (Beyotime, China), which is based on spectrophotometric methods. The activity of iNOS was measured at 530 nm according to the manufacturer’s instructions.

### ROS measurement

Measurement of ROS was performed as previously reported [64]. Briefly, samples were the same as the above iNOS detection. Samples were lysed using a bullet-blender (Bullet Blender, Next Advance, USA) and then centrifuged at 12,000 g for 10 min. After centrifugation, supernatants were collected and protein concentrations were determined by Coomassie-blue method. For ROS quantification by fluorescence, samples were incubated with 10 mM carboxy-H_2_DCFDA (Sigma, USA) at 37°C for 30 min in the dark and analyzed by a microplate reader (BioTek, synergy 2, USA) at an excitation and emission wavelength of 495 and 525 nm, respectively.

### Phagocytosis assay

Macrophages were separated from head kidney of Nile tilapia as previously described [65]. Briefly, the head kidneys were removed aseptically and pressed through an 80 µm sterile steel mesh and re-suspended in L-15 medium (Gibico, USA) supplemented with 10% fetal bovine serum (FBS) (Gibico, USA) and 1% penicillin/streptomycin (Sigma, USA). The cell suspensions were layered onto a 54%/31% discontinuous percoll (Sigma, USA) density gradient and centrifuged at 400 × g at 4°C for 40 min. The cells were collected and cultured at 25°C for 24 h. After washing and removing the non-adherent cells, the cells were diluted to 5 × 10^6^ cells/mL and resuspended in the low serum L-15 medium with 0.5% FBS for overnight starvation stressing. Then, the medium was replaced into the non-serum L-15 and then prepared for phagocytosis. Phagocytosis assays were referred to methods already available in the laboratory [20]. Briefly, the cells were deprived of serum overnight and then incubated alone or additively with 20 mM of taurine or malic acid at 25°C for 4 h. After pretreating, FITC-conjugated *V. alginolyticus* cells were centrifuged onto macrophages at a multiplicity of infection of 100 in the indicated medium. Then, the plates were placed at 25°C for 1.5 h. After infection, the monocytes/macrophages were washed with cold PBS to stop Phagocytosis. Cells were harvested and analyzed by flow cytometer (CytoFLEX, Beckman Coulter Ltd., USA).

## References

[1] Economopoulou A, Chochlakis D, Almpan MA, et al. Environmental investigation for the presence of *Vibrio* species following a case of severe gastroenteritis in a touristic island. Environ Sci Pollut Res Int. 2017;24:4835–4840. 27987123 10.1007/s11356-016-8231-7

[2] Oberbeckmann S, Wichels A, Wiltshire KH, et al. Occurrence of *Vibrio parahaemolyticus* and *Vibrio alginolyticus* in the German Bight over a seasonal cycle. Antonie Van Leeuwenhoek. 2011;100:291–307. 21598011 10.1007/s10482-011-9586-x

[3] Lun JS, Liu D, Liu TK, et al. Evaluation of outer membrane protein U (OmpU) as a novel capture target of *Vibrio parahaemolyticus* and rapid detection of acute hepatopancreatic necrosis disease (AHPND) using PCR combined with immunomagnetic separation. Aquaculture. 2018;485:225–232.

[4] Mohamad N, Mohd Roseli FA, Azmai MNA, et al. Natural concurrent infection of *Vibrio harveyi* and *V. alginolyticus* in cultured hybrid groupers in Malaysia. J Aquat Anim Health. 2019;31:88–96. 30536485 10.1002/aah.10055

[5] Rojas R, Miranda CD, Romero J, et al. Isolation and pathogenic characterization of *Vibrio bivalvicida* associated with a massive larval mortality event in a commercial hatchery of scallop *Argopecten purpuratus* in Chile. Front Microbiol. 2019;10:855. 10.3389/fmicb.2019.00855 PMC6524457 31133994

[6] Wang Q, Ji W, Xu Z. Current use and development of fish vaccines in China. Fish Shellfish Immunol. 2020;96:223–234. 31821845 10.1016/j.fsi.2019.12.010

[7] Li H, Chu X, Peng B, et al. DNA shuffling approach for recombinant polyvalent OmpAs against *V*. alginolyticus and *E. tarda* infections. Fish Shellfish Immunol. 2016;58:508–513. 27697557 10.1016/j.fsi.2016.09.058

[8] Adams A. Progress, challenges and opportunities in fish vaccine development. Fish Shellfish Immunol. 2019;90:210–214. 31039441 10.1016/j.fsi.2019.04.066

[9] Ben Hamed S, Tavares Ranzani-Paiva MJ, Tachibana L, et al. Fish pathogen bacteria: adhesion, parameters influencing virulence and interaction with host cells. Fish Shellfish Immunol. 2018;80:550–562. 29966687 10.1016/j.fsi.2018.06.053

[10] Kim S, Aga DS. Potential ecological and human health impacts of antibiotics and antibiotic-resistant bacteria from wastewater treatment plants. J Toxicol Environ Health B Crit Rev. 2007;10:559–573. 18049923 10.1080/15287390600975137

[11] Zhang Q, Dick WA. Growth of soil bacteria, on penicillin and neomycin, not previously exposed to these antibiotics. Sci Total Environ. 2014;493:445–453. 24956077 10.1016/j.scitotenv.2014.05.114

[12] Peng B, Li H, Peng XX. Functional metabolomics: from biomarker discovery to metabolome reprogramming. Protein Cell. 2015;6:628–637. 26135925 10.1007/s13238-015-0185-x PMC4537470

[13] Cheng ZX, Guo C, Chen ZG, et al. Glycine, serine and threonine metabolism confounds efficacy of complement-mediated killing. Nat Commun. 2019;10:3325. 31346171 10.1038/s41467-019-11129-5 PMC6658569

[14] Gong Q, Yang D, Jiang M, et al. L-aspartic acid promotes fish survival against Vibrio alginolyticus infection through nitric oxide-induced phagocytosis. Fish Shellfish Immunol. 2019;97:359–366. 31866447 10.1016/j.fsi.2019.12.061

[15] Yang JH, Bhargava P, McCloskey D, et al. Antibiotic-induced changes to the host metabolic environment inhibit drug efficacy and alter immune function. Cell Host Microbe. 2017;22:757–765. 29199098 10.1016/j.chom.2017.10.020 PMC5730482

[16] Stokes JM, Lopatkin AJ, Lobritz MA, et al. Bacterial metabolism and antibiotic efficacy. Cell Metab. 2019;30:251–259. 31279676 10.1016/j.cmet.2019.06.009 PMC6990394

[17] Zhang S, Wang J, Jiang M, et al. Reduced redox-dependent mechanism and glucose-mediated reversal in gentamicin-resistant *Vibrio alginolyticus*. Environ Microbiol. 2019;21:4724–4739. Epub ahead of print. 10.1111/1462-2920.14811 31595636

[18] Liu SR, Peng XX, Li H. Metabolic mechanism of ceftazidime resistance in *Vibrio alginolyticus*. Infect Drug Resist. 2019;12:417–429. 30863124 10.2147/IDR.S179639 PMC6388739

[19] Zeng ZH, Du CC, Liu SR, et al. Glucose enhances tilapia against *Edwardsiella tarda* infection through metabolome reprogramming. Fish Shellfish Immunol. 2017;61:34–43. 27965164 10.1016/j.fsi.2016.12.010

[20] Chen XH, Liu SR, Peng B, et al. Exogenous L-valine promotes phagocytosis to kill multidrug-resistant bacterial pathogens. Front Immunol. 2017;8:207. 28321214 10.3389/fimmu.2017.00207 PMC5337526

[21] Cheng ZX, Yang MJ, Peng B, et al. The depressed central carbon and energy metabolisms is associated to the acquisition of levofloxacin resistance in *Vibrio alginolyticus*. J Proteomics. 2018;181:83–91. 29627625 10.1016/j.jprot.2018.04.002

[22] Xu D, Wang J, Guo C, et al. Elevated biosynthesis of palmitic acid is required for zebrafish against *Edwardsiella tarda* infection. Fish Shellfish Immunol. 2019;92:508–518. 31247319 10.1016/j.fsi.2019.06.041

[23] Chen XH, Zhang BW, Li H, et al. Myo-inositol improves the host’s ability to eliminate balofloxacin-resistant *Escherichia coli*. Sci Rep. 2015;5:10720. 26030712 10.1038/srep10720 PMC5377236

[24] Cheng ZX, Ma YM, Li H, et al. N-acetylglucosamine enhances survival ability of tilapias infected by *Streptococcus iniae*. Fish Shellfish Immunol. 2014;40:524–530. 25120218 10.1016/j.fsi.2014.08.008

[25] Ma YM, Yang MJ, Wang S, et al. Liver functional metabolomics discloses an action of L-leucine against *Streptococcus iniae* infection in tilapias. Fish Shellfish Immunol. 2015;45:414–421. 25957884 10.1016/j.fsi.2015.04.037

[26] Zhao XL, Han Y, Ren ST, et al. L-proline increases survival of tilapias infected by *Streptococcus agalactiae* in higher water temperature. Fish Shellfish Immunol. 2015;44:33–42. 25659229 10.1016/j.fsi.2015.01.025

[27] Jiang M, Gong QY, Lai SS, et al. Phenylalanine enhances innate immune response to clear ceftazidime-resistant *Vibrio alginolyticus* in *Danio rerio*. Fish Shellfish Immunol. 2019;84:912–919. 30389644 10.1016/j.fsi.2018.10.071

[28] Yang MJ, Cheng ZX, Jiang M, et al. Boosted TCA cycle enhances survival of zebrafish to *Vibrio alginolyticus* infection. Virulence. 2018;9:634–644. 29338666 10.1080/21505594.2017.1423188 PMC5955478

[29] Yamada T, Letunic I, Okuda S, et al. iPath2.0: interactive pathway explorer. Nucleic Acids Res. 2011;39:W412–W415. 21546551 10.1093/nar/gkr313 PMC3125749

[30] Zheng L, Xu Y, Lu J, et al. Variant innate immune responses of mammary epithelial cells to challenge by *Staphylococcus aureus, Escherichia coli* and the regulating effect of taurine on these bioprocesses. Free Radic Biol Med. 2016;96:166–180. 27107770 10.1016/j.freeradbiomed.2016.04.022

[31] Lin CJ, Chiu CC, Chen YC, et al. Taurine attenuates hepatic inflammation in chronic alcohol-fed rats through inhibition of TLR4/MyD88 signaling. J Med Food. 2015;18:1291–1298. 26090712 10.1089/jmf.2014.3408 PMC4685501

[32] Miao J, Zheng L, Zhang J, et al. The effect of taurine on the toll-like receptors/nuclear factor kappa B (TLRs/NF-κB) signaling pathway in *Streptococcus uberis*-induced mastitis in rats. Int Immunopharmacol. 2011;11:1740–1746. 21745598 10.1016/j.intimp.2011.06.008

[33] Guo Z, Lin Y, Wang X, et al. The protective efficacy of four iron-related recombinant proteins and their single-walled carbon nanotube encapsulated counterparts against *Aeromonas hydrophila* infection in zebrafish. Fish Shellfish Immunol. 2018;82:50–59. 30086377 10.1016/j.fsi.2018.08.009

[34] Cheng ZX, Chu X, Wang SN, et al. Six genes of *ompA* family shuffling for development of polyvalent vaccines against *Vibrio alginolyticus* and *Edwardsiella tarda*. Fish Shellfish Immunol. 2018;75:308–315. 29438846 10.1016/j.fsi.2018.02.022

[35] Defoirdt T, Sorgeloos P, Bossier P. Alternatives to antibiotics for the control of bacterial disease in aquaculture. Curr Opin Microbiol. 2011;14:251–258. 21489864 10.1016/j.mib.2011.03.004

[36] Yang J, Zeng ZH, Yang MJ, et al. NaCl promotes antibiotic resistance by reducing redox states in *Vibrio alginolyticus*. Environ Microbiol. 2018;20:4022–4036. 30307102 10.1111/1462-2920.14443

[37] Su YB, Peng B, Li H, et al. Pyruvate cycle increases aminoglycoside efficacy and provides respiratory energy in bacteria. Proc Natl Acad Sci U S A. 2018;115:E1578–E1587. 29382755 10.1073/pnas.1714645115 PMC5816162

[38] Peng B, Su YB, Li H, et al. Exogenous alanine and/or glucose plus kanamycin kills antibiotic-resistant bacteria. Cell Metab. 2015;21:249–262. 25651179 10.1016/j.cmet.2015.01.008

[39] Haller T, Lackner R. Screening for organic acids in fish tissues with special reference to the distribution of taurine in *Rutilus rutilus* L. Fish Physiol Biochem. 1987;3:145–149. 24233442 10.1007/BF02180416

[40] Liu CL, Watson AM, Place AR, et al. Taurine biosynthesis in a fish liver cell line (ZFL) adapted to a serum-free medium. Mar Drugs. 2017;15:147. 10.3390/md15060147 PMC5484097 28587087

[41] Dong J, Cheng R, Yang Y, et al. Effects of dietary taurine on growth, non-specific immunity, anti-oxidative properties and gut immunity in the Chinese mitten crab *Eriocheir sinensis*. Fish Shellfish Immunol. 2018;82:212–219. 30125701 10.1016/j.fsi.2018.08.029

[42] Shen G, Wang S, Dong J, et al. Metabolic effect of dietary taurine supplementation on Grouper (*Epinephelus coioides*): A ^1^H-NMR-based metabolomics study. Molecules. 2019;24:2253. 10.3390/molecules24122253 PMC6630984 31212947

[43] Kortner TM, Penn MH, Bjӧrkhem I, et al. Bile components and lecithin supplemented to plant based diets do not diminish diet related intestinal inflammation in Atlantic salmon. BMC Vet Res. 2016;12:190. 27604133 10.1186/s12917-016-0819-0 PMC5015236

[44] Lu J, Shi Y, Cai S, et al. Metabolic responses of *Haliotis diversicolor* to *Vibrio parahaemolyticus* infection. Fish Shellfish Immunol. 2017;60:265–274. 27890800 10.1016/j.fsi.2016.11.051

[45] Shao Y, Li C, Ou C, et al. Divergent metabolic responses of *Apostichopus japonicus* suffered from skin ulceration syndrome and pathogen challenge. J Agric Food Chem. 2013;61:10766–10771. 24127639 10.1021/jf4038776

[46] Sartori T, Dos Santos GG, Nogueira-Pedro A, et al. Effects of glutamine, taurine and their association on inflammatory pathway markers in macrophages. Inflammopharmacology. 2018;26:829–838. 29052795 10.1007/s10787-017-0406-4

[47] Sanchez LM, Cheng AT, Warner CJ, et al. Biofilm formation and detachment in gram-negative pathogens is modulated by select bile acids. PLoS One. 2016;11:e0149603. 26992172 10.1371/journal.pone.0149603 PMC4798295

[48] Dai B, Zhang J, Liu M, et al. The role of (Ca2+) mediated signaling pathways on the effect of taurine against *Streptococcus uberis* infection. Vet Microbiol. 2016;192:26–33. 27527761 10.1016/j.vetmic.2016.06.008

[49] Zhai N, Wang H, Chen Y, et al. Taurine attenuates OTA-promoted PCV2 replication through blocking ROS-dependent autophagy via inhibiting AMPK/mTOR signaling pathway. Chem Biol Interact. 2018;296:220–228. 30332612 10.1016/j.cbi.2018.10.005

[50] Kim SH, Zhong X, Kim W, et al. Taurine chloramine potentiates phagocytic activity of peritoneal macrophages through up-regulation of dectin-1 mediated by heme oxygenase-1-derived carbon monoxide. Faseb J. 2018;32:2246–2257. 29247123 10.1096/fj.201700817R

[51] Tsai YC, Tsai TF. Anti-interleukin and interleukin therapies for psoriasis: current evidence and clinical usefulness. Ther Adv Musculoskelet Dis. 2017;9:277–294. 29344110 10.1177/1759720X17735756 PMC5764033

[52] Jung IH, Choi JH, Chung YY, et al. Predominant activation of JAK/STAT3 pathway by interleukin-6 is implicated in hepatocarcinogenesis. Neoplasia. 2015;17:586–597. 26297436 10.1016/j.neo.2015.07.005 PMC4547407

[53] Wang X, Wei L, Wang Y, et al. Evaluation of development, locomotor behavior, oxidative stress, immune responses and apoptosis in developing zebrafish (*Danio rerio*) exposed to TBECH (tetrabromoethylcyclohexane). Comp Biochem Physiol C Toxicol Pharmacol. 2019;217:106–113. 30528700 10.1016/j.cbpc.2018.12.004

[54] Jian L, Sun L, Li C, et al. Interleukin-21 enhances Toll-like receptor 2/4-mediated cytokine production via phosphorylation in the STAT3, Akt and p38 MAPK signaling pathways in human monocytic THP-1 cells. Scand J Immunol. 2019;89:e12761. 30977163 10.1111/sji.12761

[55] Szondy Z, Pallai A. Transmembrane TNF-alpha reverse signaling leading to TGF-beta production is selectively activated by TNF targeting molecules: therapeutic implications. Pharmacol Res. 2017;115:124–132. 27888159 10.1016/j.phrs.2016.11.025

[56] Cheong SH, Yang HW, Ko EY, et al. Anti-inflammatory effects of trans-1,3-diphenyl-2,3-epoxypropane-1-one in zebrafish embryos in vivo model. Fish Shellfish Immunol. 2016;50:16–20. 26796815 10.1016/j.fsi.2016.01.018

[57] Wang T, Yan J, Xu W, et al. Characterization of cyclooxygenase-2 and its induction pathways in response to high lipid diet-induced inflammation in *Larmichthys crocea*. Sci Rep. 2016;6:19921. 26830811 10.1038/srep19921 PMC4735279

[58] Pezzatti J, González-Ruiz V, Codesido S, et al. A scoring approach for multi-platform acquisition in metabolomics. J Chromatogr A. 2019;1592:47–54. 30685186 10.1016/j.chroma.2019.01.023

[59] Zhu W, Zhang H, Li X, et al. Cold adaptation mechanisms in the ghost moth *Hepialus xiaojinensis*: metabolic regulation and thermal compensation. J Insect Physiol. 2016;85:76–85. 26585102 10.1016/j.jinsphys.2015.11.008

[60] Chong J, Xia J. MetaboAnalystR: an R package for flexible and reproducible analysis of metabolomics data. Bioinformatics. 2018;34:4313–4314. 29955821 10.1093/bioinformatics/bty528 PMC6289126

[61] Chong J, Soufan O, Li C, et al. MetaboAnalyst 4.0: towards more transparent and integrative metabolomics analysis. Nucleic Acids Res. 2018;46:W486–W494. 29762782 10.1093/nar/gky310 PMC6030889

[62] Wang Y, Wang X, Ali F, et al. Comparative extracellular proteomics of *Aeromonas hydrophila* reveals iron-regulated secreted proteins as potential vaccine candidates. Front Immunol. 2019;10:256. 30833947 10.3389/fimmu.2019.00256 PMC6387970

[63] Livak KJ, Schmittgen TD. Analysis of relative gene expression data using real-time quantitative PCR and the 2(-Delta Delta C(T)) method. Methods. 2001;25:402–408. 11846609 10.1006/meth.2001.1262

[64] Ye JZ, Su YB, Lin XM, et al. Alanine enhances aminoglycosides-induced ROS production as revealed by proteomic analysis. Front Microbiol. 2018;9:29. 29441044 10.3389/fmicb.2018.00029 PMC5797687

[65] Mu L, Yin X, Yang Y, et al. Functional characterization of a mannose-binding lectin (MBL) from Nile tilapia (*Oreochromis niloticus*) in non-specific cell immunity and apoptosis in monocytes/macrophages. Fish Shellfish Immunol. 2019;87:265–274. 30654028 10.1016/j.fsi.2019.01.019

