# Supplementary material for: Malate enhances survival of zebrafish against Vibrio alginolyticus infection in the same manner as taurine
Source: Virulence. 2020 Apr 21;11(1):349–64. doi: 10.1080/21505594.2020.1750123 (PMC7199751; doi:10.1080/21505594.2020.1750123)
Supplement: Supplemental Material [file kvir-11-01-1750123-s001.docx]

**Supplementary** **Table 1. Primers for qRT-PCR**

| Gene | Primer | Sequence (5'-3') |
| --- | --- | --- |
| *gyrB* | Forward | ATTGAGAACCCGACAGAAGCGAAG |
|  | Reverse | CCTAATGCGGTGATCAGTGTTACT |
| *β-actin* | Forward | ACCCAGACATCAGGGAGTG |
|  | Reverse | CATCCCAGTTGGTCACAATAC |
| *ptgs-2* | Forward | TTCTTCGCCCAGCATTTCTC |
|  | Reverse | AATGTGCCCCAGATCCACT |
| *tnf-a* | Forward | ATAAGACCCAGGGCAATCAAC |
|  | Reverse | CAGAGTTGTATCCACCTGTTAAATG |
| *c3b* | Forward | TGTGACCCGCTGTATGTTCT |
|  | Reverse | TTGGCTGGGAAGTTCTTCAC |
| *Il-1b* | Forward | TGGACTTCGCAGCACAAAATG |
|  | Reverse | GTTCACTTCACGCTCTTGGATG |
| *Il-10* | Forward | CTCTGCTCACGCTTCTTC |
|  | Reverse | TCATCGTTGGACTCATAAAAC |
| *Il-6* | Forward | ATCCGCTCAGAAAACAGTGCT |
|  | Reverse | GTCGCCAAGGAGACTCTTTAC |
| *Il-8* | Forward | CACGCTGTCGCTGCATTG |
|  | Reverse | GTCATCAAGGTGGCAATGATCTC |
| *Il-21* | Forward | CTAAAGTGCTGCACCTGTCAG |
|  | Reverse | TTGCACTGAGCTTTCTGTGTC |
| *arg2* | Forward | GACTTTCAAATCTTGACTACCCTGT |
|  | Reverse | ACTGAGCCAATCGCTAAGCTG |
| *otc* | Forward | AGACACGGCCAGGGTTCTTT |
|  | Reverse | TGAGCCATAGTGTTCCTGTAGTGTT |
| *nos2b* | Forward | GAATCACTGCTCTGTCGACTCTT |
|  | Reverse | CCTGCGAAAATCCCCACAT |
| *asl* | Forward | GCATACAGCACTGGGAGCAGT |
|  | Reverse | CTGTAAGTCTTTGTTGTACGTGCTT |
| *agxta* | Forward | AATGAGGAAATTGAGAAGGCATTAA |
|  | Reverse | GAAAATATCAATACCTTGTTCATCCA |
| *agxtb* | Forward | TGCACTCGGAAATGTTCGG |
|  | Reverse | CTTTGCACCCATGCGCTC |
| *agxt2* | Forward | GATGAGGTCCAGACTGGCTTTG |
|  | Reverse | TGGCAATTTCTGCAGTCGTGA |
| *hao1* | Forward | CTCTCATTTGAGGATGGCGAAT |
|  | Reverse | CTTCTTTAGCGTCTTCAGCTGTCA |
| *hao2* | Forward | ATAAAAGGATTCGCTTGAGGCC |
|  | Reverse | TCAACGCCTCAGTAGCTCTGG |
| *aco1* | Forward | AAAGACCCCAGATATGAGCAGCT |
|  | Reverse | ACTGCAGGTACACCCGTGAAG |
| *aco2* | Forward | TCGCCATTACAAAAAGAACAACATT |
|  | Reverse | CCCTGTTTCTTCAAGTTTGTTTCAT |
| *shmt1* | Forward | CAATGATCCTGAGGTGTTTGACAT |
|  | Reverse | GCCTCCATAATACCTCTGGCC |
| *psat1* | Forward | GTGGACGTGTCAAAGTTTGGTC |
|  | Reverse | TAAATACTGAAGCATGGAGGCG |
| *psph* | Forward | ACCCGGCATCAGGGAGCT |
|  | Reverse | GGCGTACTCTCCATTGAAGTAGAAC |
| *gad1a* | Forward | GGAATGAAGATATGTGGGTTCTTG |
|  | Reverse | AGGAAGCAGATCTCTTGCATACAG |
| *adob* | Forward | CATCGCTCCTCCCGTCACAT |
|  | Reverse | ATCCAGGCGGTCGAAACAAC |
| *csad* | Forward | GGCAAAATCCCAAGACGCTG |
|  | Reverse | CGAGTTTGCTCTTTCTATTCCTGC |
| *phgdh* | Forward | GCAGAGATTCGGAATTATGATGGT |
|  | Reverse | TCCGCTTGGAGTGTTCATGAC |
| *cdo1* | Forward | ACACGGCAGCAGCATCCAT |
|  | Reverse | GGAGTCCCAGAGAGTCGTTGAT |
